# Divergent Behavioral Phenotypes and Transcriptomic Reprogramming in *Lymantria dispar* Larvae Infected by Virus, Bacterium and Fungus

**DOI:** 10.3390/biology15080656

**Published:** 2026-04-21

**Authors:** Lin-Bo Zhai, Ya-Jie Wang, Jiang-Bo Zhang, Dun Wang

**Affiliations:** 1 Key Laboratory of Plant Protection Resources and Pest Management of Ministry of Education, College of Plant Protection, Northwest A&F University, Yangling 712100, China; linbozhai@foxmail.com (L.-B.Z.); wangshiyishi@outlook.com (Y.-J.W.); 2 College of Life Science, Northwest A&F University, Yangling 712100, China; 3 Key Laboratory of Integrated Pest Management on the Loess Plateau of Ministry of Agriculture and Rural Affairs, Northwest A&F University, Yangling 712100, China

**Keywords:** behavioral manipulation, baculovirus, *Lymantria dispar*, tree-top disease, comparative transcriptomics, host–pathogen interaction

## Abstract

Pathogen manipulation of host behavior is a widespread evolutionary strategy to enhance its transmission, yet whether different pathogens elicit distinct behavioral and molecular responses in the same host remains poorly understood. We performed parallel behavioral assays and comparative transcriptomic analyses on third-instar *Lymantria dispar* larvae infected with *Lymantria dispar* multiple nucleopolyhedrovirus (LdMNPV, virus), *Staphylococcus aureus* (bacterium) and *Metarhizium anisopliae* (fungus). Climbing height was recorded over 72 h post-infection, and gene expression pattern was profiled using RNA-seq at 72 h. Only LdMNPV infection induced significant, sustained upward climbing behavior among the three pathogen infection groups. All three pathogens activated Toll and IMD immune pathways, but LdMNPV triggered substantially broader transcriptomic reprogramming. Notably, the virus specifically upregulated multiple energy metabolism pathways (nicotinate/nicotinamide metabolism, pyruvate metabolism, TCA cycle and oxidative phosphorylation) and the neuroactive ligand-receptor interaction pathway—a pattern absent in bacterial and fungal infections. LdMNPV drove tree-top disease through a virus-specific, multi-system manipulation strategy that couples metabolic activation with neural signaling modulation. This comparative study reveals fundamental differences in behavioral manipulation across pathogen kingdoms and provides candidate pathways for functional validation.

## Simple Summary

Pathogens can sometimes alter the behavior of infected animals to enhance their own spread. In this study, the climbing behavior was investigated in gypsy moth larvae infected with a virus, a bacterium and a fungus, respectively. It was found that only the viral pathogen *Lymantria dispar* multiple nucleopolyhedrovirus (LdMNPV) caused larvae to climb higher significantly, whereas the bacterium and fungus did not. By analyzing gene expression patterns, we discovered that the virus specifically activated genes related to energy production and nerve signaling, while the bacterium and fungus did not. These findings suggest that the virus employs a unique strategy to increase host activity and height-seeking behavior, likely to improve its transmission to new hosts. This work enhances our understanding of how different pathogens interact with their hosts and may shed light on the study of animal behavioral regulation during pathogen–host interactions.

## 1. Introduction

*Lymantria dispar* is a globally distributed, highly polyphagous forest pest capable of feeding on more than 500 host plants and causing substantial ecological and economic losses, making it an important target for microbial control and host–pathogen research [1,2]. Throughout the long coevolutionary history of hosts and pathogens, diverse pathogens have evolved the remarkable ability to manipulate host behavior to promote their own transmission—a phenomenon termed “parasitic manipulation” or the “extended phenotype” [3,4]. For example, rabies virus affects the host central nervous system, inducing increased aggression to facilitate bite transmission [5]; *Toxoplasma gondii* weakens the innate aversion of rodents to predator odors, increasing their probability of being preyed upon by the definitive host [6]; and the renowned entomopathogenic fungus *Ophiocordyceps* precisely manipulates ants to climb to positions favorable for spore dispersal and die while fixed in place [7,8,9]. *Hairworm* (Nematomorpha) exploit polarized light reflected from water surfaces at night to attract terrestrial hosts such as crickets and mantises, causing them to jump into water, thereby completing the aquatic stage of their life cycle [10,11,12].

Baculoviridae is a large family of insect-specific viruses, divided into four genera [13]. Within this family, nucleopolyhedroviruses (Alphabaculovirus) and granuloviruses (Betabaculovirus) cause lethal infections in lepidopteran hosts [14]. The “tree-top disease” induced by baculovirus infection in lepidopteran larvae is a classic model for studying viral behavioral manipulation [15]. Infected larvae exhibit sustained upward climbing, eventually dying at elevated positions; their cadavers liquefy, releasing viral occlusion bodies that disperse aerially, greatly enhancing transmission efficiency [16,17]. Although previous studies have identified several viral genes associated with tree-top disease—such as the ecdysteroid UDP-glucosyltransferase gene (*egt*) and the protein tyrosine phosphatase gene (*ptp*) [18,19]—the complete mechanisms by which viruses integrate and reprogram host endogenous gene networks to ultimately drive behavioral change remain unclear. In *L. dispar* specifically, transcriptomic analyses across the baculovirus-induced hyperactive stage revealed dynamic host gene-expression reprogramming before terminal climbing [20]. Recent studies have begun to investigate the involvement of environmental signals (e.g., light) [21,22], host hormonal pathways [23], metabolic pathways [24,25], the immune system [26,27], phototransduction pathways [28,29], and the nervous system [30], suggesting that behavioral manipulation is a complex process involving multisystem interactions.

Certain entomopathogenic fungi have convergently evolved the capacity to manipulate host behaviors. For instance, *Cordyceps militaris* secretes a host-mimetic trehalase (CmTreH1) that degrades trehalose in the silkworm hemolymph, inducing a state of perceived starvation and promoting hyperphagia—providing the first mechanistic evidence for fungal manipulation of host feeding behavior [31]. Fungi that cause “summit disease” are hypothesized to target host circadian rhythms, molting, and even neuronal structures such as mushroom bodies, suggesting a multisystem manipulation strategy parallel to that of baculoviruses [32]. Our previous work showed that *M. anisopliae* promotes tree-top disease behavior during early LdMNPV infection in *L. dispar* but inhibits it during late stages [33].

In stark contrast, evidence for bacteria actively manipulating host behavior to enhance their own transmission remains remarkably scarce. Although recent studies have demonstrated that gut microbes can influence host social behavior (e.g., aggregation in *Caenorhabditis elegans*) or feeding decisions via metabolic byproducts, these effects appear to be indirect consequences of commensal–host cometabolism rather than adaptive manipulation strategies evolved specifically for parasitic benefit [34]. Indeed, in many insect–bacterium systems, the host actively suppresses bacterial pathogenicity and drives the symbiont toward commensalism [35], further highlighting the difficulty for bacteria to establish sustained behavioral control.

Previous research has shown that following LdMNPV infection in gypsy moth larvae, host symbiotic bacteria exhibit significant shifts: increased abundance of Gram-negative bacteria correlates with elevated Imd pathway expression, while decreased abundance of Gram-positive bacteria correlates with reduced Toll pathway expression [36]. In addition to viral infection, temporospatial transcriptomic studies have characterized the immune response of *L. dispar* to entomopathogenic fungi, revealing strong tissue- and time-dependent regulation of antifungal immunity [37,38]. Species-specific studies have also documented large-scale transcriptomic responses of *L. dispar* to bacterial challenge and age-dependent antiviral resistance to LdMNPV, indicating that this host already provides a substantial framework for comparative pathogen-response analyses [39].

This disparity raises a fundamental evolutionary and mechanistic question: Do different classes of pathogens elicit fundamentally distinct behavioral phenotypes and global gene expression regulation patterns when infecting the same host? To address this question, we used *L. dispar* as a model and systematically compared larval behavioral phenotypes and transcriptomic responses following infection with LdMNPV (a virus), *S. aureus* (a Gram-positive bacterium), and *M. anisopliae* (an entomopathogenic fungus). This study provides new insights into the molecular mechanisms and evolutionary strategies of pathogen behavioral manipulation from a comparative biological perspective.

## 2. Materials and Methods

### 2.1. Insect Rearing and Pathogens

*L. dispar* egg masses were collected from the vicinity of the Inner Mongolia Forestry Department and transported to the laboratory incubator (Model SPX-420B, Shanghai Nanrong Laboratory Equipment Co., Ltd., Shanghai, China) for rearing. Larvae were maintained in climate-controlled chambers at 25 ± 1 °C and 60 ± 5% relative humidity, under a 14-h light:10-h dark photoperiod, and fed an artificial diet provided by the Beijing Academy of Forestry Sciences.

The LdMNPV isolate was maintained in our laboratory stock. *S. aureus* was kindly provided by the laboratory of Professor Zhiqiang Lü (Northwest A&F University). The *M. anisopliae* fungal strain was collected from Hualong Mountain, Zhenping County, Shaanxi Province, and subsequently isolated.

The *S. aureus* strain was routinely maintained on Nutrient Agar (NA) broth and stored at −2 °C. Prior to experimentation, the strain was streaked onto NA plates and incubated overnight at 37 °C (approximately 24 h). Bacterial colonies were harvested and resuspended in 1% sterile saline solution with serial dilutions. The concentration of bacterial suspensions was determined by measuring optical density at 600 nm (OD600) using a spectrophotometer (Model NanoDrop ND-1000, Thermo Fisher Scientific, Waltham, MA, USA) and converted to colony forming units per milliliter (CFU/mL) according to a standard curve. Final working solutions were diluted to an infection concentration of 2.6 × 10^5^ CFU/μL with 1% sterile saline.

The *M. anisopliae* strains were activated and cultured on Malt Extract Agar (MEA) plates at room temperature for 4 weeks. For conidial suspension preparation, mature spores were scraped from culture plates using a sterile spatula into sterile centrifuge tubes. Sterile solution containing 0.1% Tween 20 was added, and the mixture was vortexed for 30 s to ensure adequate spore dispersion. Spore concentration was determined using a hemocytometer (Model TEK-2000, Jiangxi Tecom Science & Technology Co., Ltd., Nanchang, China) and adjusted to a working concentration of 2 × 10^7^ spores/mL.

The LdMNPV occlusion bodies (OBs) were amplified by infecting third-instar *L. dispar* larvae. Virus-killed larvae were homogenized in 1 × phosphate-buffered saline (PBS) and filtered through multiple layers of gauze to remove tissue debris. The filtrate was centrifuged at 6000 rpm for 3 min at 4 °C, and the pellet was collected. The viral pellet was resuspended and diluted to a working concentration of 1 × 10^8^ OBs/μL with ddH_2_O, aliquoted, and stored at −20 °C for subsequent use.

Preparation protocols for three pathogens followed previously peer-reviewed methods [36,40,41].

### 2.2. Infection Assay and Behavioral Observation

Newly molted third-instar larvae were starved for 24 h prior to treatment. The control group (CK) received 2 μL of 10% (*w*/*v*) sucrose solution via droplet feeding. Pathogen treatment groups were infected as follows: (1) the bacterial infection group (Sa) was fed 2 μL of *S. aureus* suspension (2.6 × 10^5^ CFU/μL); (2) the fungal infection group (LJJ3) was immersed in *M. anisopliae* spore suspension (2 × 10^7^ spores/mL); and (3) the viral infection group (NPV) was fed 2 μL of LdMNPV suspension (1 × 10^8^ OBs/μL).

Following treatment, larvae from each group were transferred into a vertical cylindrical climbing assay apparatus (60 cm in height × 6 cm in diameter) [18], which was illuminated from above only. Each apparatus contained 20 larvae from the same treatment group, and three biological replicates were established for each group. Larvae were maintained at 25 °C, 50% relative humidity, and a 14L:10D photoperiod. Climbing height assays were conducted over 72 h post-infection (hpi) at 12-h intervals. At each time point (12, 24, 36, 48, 60, and 72 hpi), larval positions were measured each 10 min period and the climbing height for that interval was calculated as the average height of 10 min period of all larvae in the mesh column but not the cumulative movement distance. Totally three replicates of measurement be taken at each time point within one hour.

### 2.3. RNA Extraction and Library Preparation for Sequencing

Total RNA was extracted from gypsy moth larvae 72 hpi with each of the three pathogens using the TRIzol method. RNA quality was assessed with a NanoDrop ND-1000 spectrophotometer (Thermo Fisher Scientific, Waltham, MA, USA) and an Agilent Bioanalyzer 2100 (Santa Clara, CA, USA). Samples meeting the quality thresholds (concentration > 50 ng/µL, RIN > 7.0, OD260/280 > 1.8, total RNA > 1 µg) were used for library construction. Poly(A)+ mRNA was enriched using oligo(dT) magnetic beads and fragmented via magnesium-based fragmentation at 94 °C for 5–7 min. First- and second-strand cDNA synthesis was performed, followed by end repair, adapter ligation, and PCR amplification to generate libraries with an average insert size of 300 bp ± 50 bp. The prepared libraries were subjected to paired-end 150 bp sequencing on an Illumina NovaSeq™ 6000 platform (San Diego, CA, USA). Each treatment group included three biological replicates, yielding a total of nine libraries.

### 2.4. Bioinformatic Analysis Pipeline

Raw sequencing reads were processed by removing adapter sequences using cutadapt and filtering out low-quality or short reads with fqtrim to obtain clean data. A de novo transcriptome analysis pipeline was then implemented, encompassing sequence assembly, functional annotation of genes, expression quantification, differential expression analysis, as well as SNP/SSR detection and CDS prediction.

To quantify the proportion of sequencing reads originating from each pathogen, clean reads (correspond to the Valid_Reads values reported in Table 1) from all samples were first aligned to the *L. dispar* reference genome (InsectBase ID: IBG_00520) using Bowtie2 v2.4.4 with the --very-sensitive parameter to generate BAM files. Subsequently, reads that failed to map to the host (i.e., unmapped reads) were extracted from these BAM files using SAMtools v1.9 with the samtools view -b -f 12 command, which retains read pairs where both ends are unmapped. These host-depleted reads were then independently aligned to the reference genomes of three pathogens using Bowtie2 v2.4.4 with the --very-sensitive parameter: LdMNPV: NCBI RefSeq assembly GCF_000846205.1; *S. aureus*: NCBI RefSeq assembly GCF_000013425.1; *M. anisopliae*: NCBI GenBank assembly GCA_013305495.1. The number of reads mapping to each pathogen genome was counted using the SAMtools command samtools view -c -F 4, which counts only the aligned (mapped) reads. The percentage of pathogen-derived reads was calculated as (number of mapped reads/Valid_Reads) × 100%.

### 2.5. Differentially Expressed Gene Analysis

In this study, transcriptome sequencing data was analyzed using the following workflow. First, Trinity 2.1.1 was used for de novo assembly of clean reads from all samples to generate a non-redundant unigene set. Functional annotation against NCBI_nr, GO, KEGG, Pfam, Swiss Prot, and eggNOG databases was performed using DIAMOND 0.9.25. Gene expression quantification was performed as TPM (transcripts per million) for expression profiling and visualization. Differential expression analysis was performed in DESeq2 1.24.0 using raw count data mapped to the unigene set, ensuring appropriate statistical analysis. Low-expression genes (those with TPM < 1 across all samples) were filtered out prior to differential expression analysis. Differentially expressed genes (DEGs) were defined as genes with |log_2_(fold change)| ≥ 1 and adjusted *p* value (FDR) < 0.05. Hierarchical clustering and volcano plots were generated using the pheatmap 1.0.12 and ggplot2 3.2.1 packages in R 3.5.2, respectively, to visualize the expression patterns and distribution of DEGs.

### 2.6. GO and KEGG Enrichment Analysis

GO functional enrichment analysis was performed on the DEGs, covering the three standard categories: molecular function, cellular component, and biological process. For KEGG pathway enrichment, a hypergeometric test was employed. The degree of enrichment was assessed using the Rich factor, and the results were visualized in a scatter plot.

### 2.7. qPCR Validation

Seven DEGs were randomly selected, and specific qPCR primers were designed for each (Table 2). Quantitative PCR was performed using the SYBR Green method. The thermal cycling protocol consisted of an initial denaturation at 95 °C for 10 min, followed by 45 cycles of 95 °C for 10 s, 60 °C for 10 s, and 72 °C for 10 s. Relative gene expression levels were calculated using the 2^−ΔΔCt^ method to validate the reliability of the transcriptomic data.

### 2.8. Statistical Analysis

To compare differences in climbing height among different pathogen infection groups and the control group at each time point, data were analyzed using a Generalized Linear Model (GLM). In the model, mean climbing height was specified as the dependent variable, and treatment group (CK, Sa, LJJ3, NPV) was included as a fixed factor, with a normal distribution family and identity link function. To account for potential heteroscedasticity, Huber-White heteroscedasticity-consistent robust standard errors were used for parameter estimation. The overall treatment group effect was evaluated using the Wald χ^2^ test. When the overall test was significant (*p* < 0.05), Dunnett’s test was subsequently performed for multiple comparisons, with each infection group (Sa, LJJ3, NPV) compared separately against the control group (CK), and *p*-values from multiple comparisons were adjusted using Dunnett’s method. All analyses were conducted in SPSS 26.0 (GENLIN procedure), and data are presented as mean ± standard error of the mean (Mean ± SEM). Significance is indicated as: *p* > 0.05 (ns), *p* < 0.05 (*), *p* < 0.01 (**), *p* < 0.001 (***), *p* < 0.0001 (****).

## 3. Results

### 3.1. Climbing Behavior Is Specifically Induced by LdMNPV Infection

**Figure 1:**
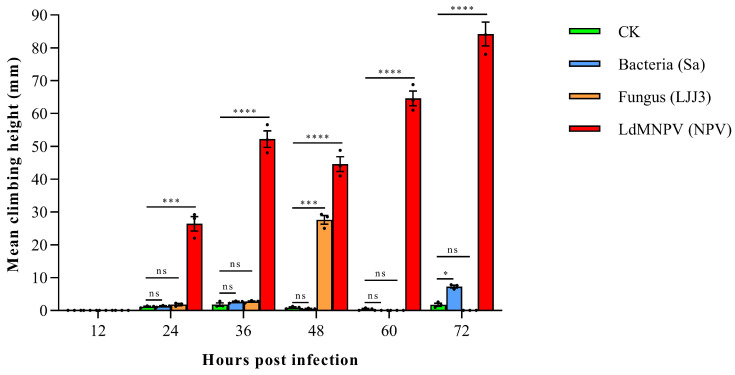
LdMNPV infection specifically induces climbing behavior in larvae. At 12 h post-infection (hpi), no climbing behavior was observed in any group (climbing height = 0 for all); therefore, statistical analysis was not performed, and the groups were considered to show no differences. For the period from 24 to 72 hpi, Generalized Linear Model (GLM) results indicated a highly significant effect of treatment group on climbing height (Wald χ^2^ = 467.61, 1015.03, 994.04, 2202.40, and 890.40, respectively; df = 3 for all; *p* < 0.001 for all). Significance is indicated as: *p* > 0.05 (ns), *p* < 0.05 (*), *p* < 0.001 (***), *p* < 0.0001 (****).

Climbing behavior assay results (Figure 1) showed that LdMNPV infection significantly induced sustained upward climbing in larvae as early as 24 h post-infection (hpi), with climbing heights highly significantly greater than the control group throughout 24–72 hpi (*p* < 0.001), exhibiting typical “tree-top disease” behavior. In contrast, S. aureus infection induced only a slight but significant increase at 72 hpi (*p* = 0.024), with no differences from the control group at other time points. *M. anisopliae* infection showed only a transient significant increase at 48 hpi (*p* < 0.001). The control group (CK) maintained consistently low climbing levels throughout the experiment with no obvious behavioral changes.

Collectively, these results demonstrate that LdMNPV-induced climbing behavior is virus-specific, whereas bacterial and fungal infections elicited only transient or subtle behavioral alterations. These findings further corroborate the conclusion that “tree-top disease” represents a virus-specific phenotype.

### 3.2. High-Quality Transcriptome Assembly and Verification of Pathogen Infection Specificity

**Figure 2:**
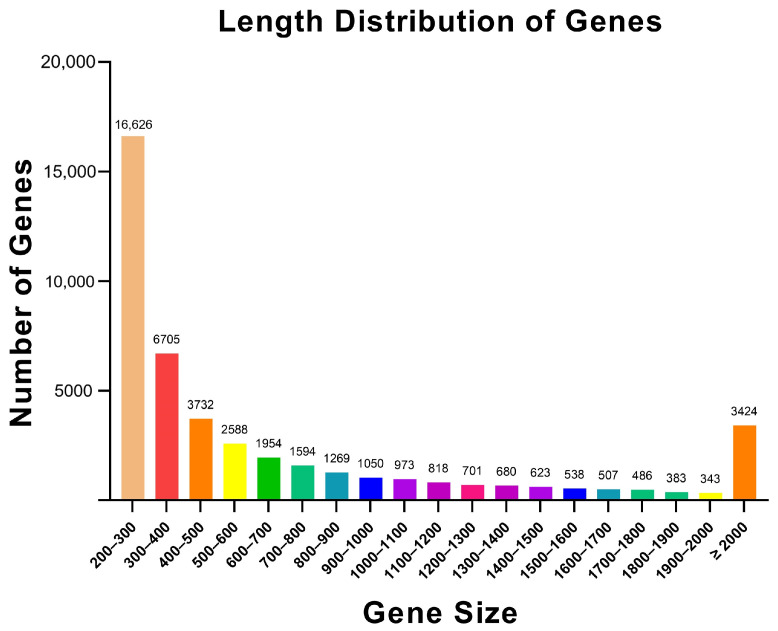
Unigene length distribution.

Transcriptome sequencing was performed on *L. dispar* larvae separately CK and infected with a virus (LdMNPV), a bacterium (*S. aureus*) and a fungus (*M. anisopliae*). After quality control of the raw data, the percentage of valid reads for each sample ranged from 97.23% to 98.21%, with Q30 scores all exceeding 94.37% (Table 1). A pooled de novo assembly of clean reads from all samples using Trinity generated 44,994 unigenes with an N50 of 1266 bp. Unigenes with lengths between 200 and 500 bp constituted the largest proportion (60.15%) of the assembly (Figure 2).

To verify infection specificity and exclude the possibility of latent virus activation, the proportion of reads mapped to the genomes of the three pathogens was quantified for each sample (Table 2). In the control group (CK), the read proportions for *S. aureus*, *M. anisopliae*, and LdMNPV were 0.0130–0.0131%, 0.1246–0.1601%, and 0.0342–0.0416%, respectively, representing the experimental background level. In the bacterial infection group (Sa), *S. aureus* read proportions increased to 3.0464–3.5972%, while *M. anisopliae* (0.0828–0.1785%) and LdMNPV (0.0446–0.0695%) remained comparable to the control background. In the fungal infection group (LJJ3), *M. anisopliae* read proportions reached 1.7052–2.1969%, whereas *S. aureus* (0.0010–0.0018%) and LdMNPV (0.0402–0.0821%) were both at background levels. In the viral infection group (NPV), LdMNPV read proportions were as high as 21.4557–25.7046%, while *S. aureus* (0.0014–0.0039%) and *M. anisopliae* (0.0820–0.1157%) were consistent with background levels (Table 1). Notably, LdMNPV read proportions in all non-viral treatment groups were ≤ 0.0821%, comparable to the control background and substantially lower than the 21.4557–25.7046% observed in the viral infection group. These results exclude the possibility of latent baculovirus activation and confirm that the observed phenotypic differences were specifically induced by the respective target pathogens.

### 3.3. Comparative Transcriptomic Analysis Reveals Pathogen-Specific Host Responses

**Figure 3:**
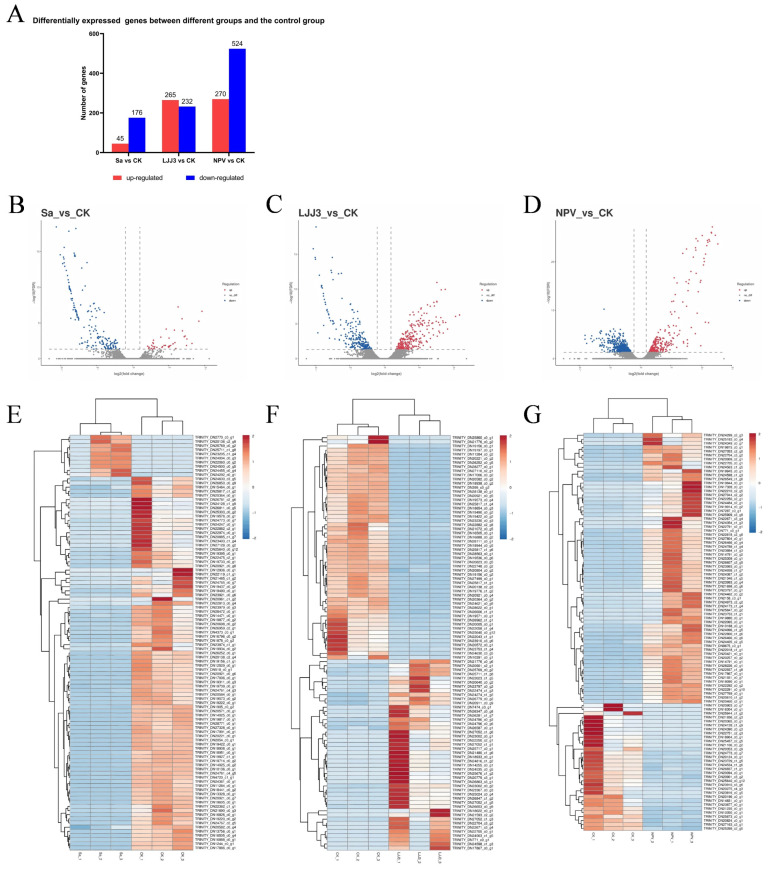
Comparative transcriptomic analysis reveals distinct host gene expression profiles in response to bacterial, fungal, and viral infections. (**A**) Statistics of differentially expressed genes (DEGs) showing up-regulated and down-regulated counts in pairwise comparisons between each infection group and the control (CK). (**B**–**D**) Volcano plots visualizing the distribution and significance of DEGs for each infection versus control: (**B**) *S. aureus* (Sa) versus CK; (**C**) *M. anisopliae* (LJJ3) versus CK; (**D**) LdMNPV (NPV) versus CK. (**E**–**G**) Hierarchical clustering heatmaps illustrating the expression patterns of identified DEGs across biological replicates for each comparison: (**E**) *S. aureus* versus CK; (**F**) *M. anisopliae* versus CK; (**G**) LdMNPV versus CK. Red and blue colors indicate high and low relative expression levels, respectively. In the volcano plots (**B**–**D**), the dashed vertical lines indicate the fold-change thresholds (log2(fold change) = ±1), and the dashed horizontal line indicates the significance threshold (FDR = 0.05; corresponding to −log10(FDR) = 1.30).

Comparisons between each infection group and the control group identified distinct sets of differentially expressed genes (DEGs). When comparing the *S. aureus* infection to the control, 45 genes were up-regulated and 176 were down-regulated. The *M. anisopliae* infection showed 265 up-regulated and 232 down-regulated genes relative to the control. Comparing the LdMNPV infection to the control revealed 270 up-regulated and 524 down-regulated genes. The overall distribution of these DEGs in each comparison was visualized in volcano plots, while hierarchical clustering heatmaps further illustrated the distinct expression patterns across different samples (Figure 3).

**Figure 4:**
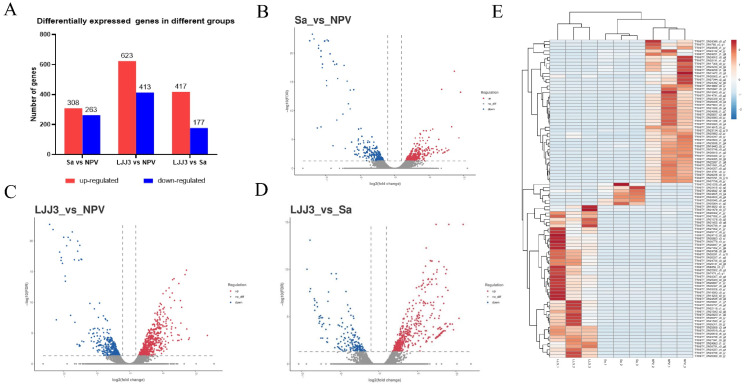
Comparative transcriptomic profiling reveals pathogen-specific host responses. (**A**) Number of differentially expressed genes (DEGs) identified in pairwise comparisons between *L. dispar* larvae infected with LdMNPV (virus), *S. aureus* (bacteria), or *M. anisopliae* (fungus). (**B**–**D**) Volcano plots visualizing the distribution and significance of DEGs for each comparison: (**B**) *S. aureus* (Sa) versus LdMNPV (NPV). (**C**) *M. anisopliae* (LJJ3) versus LdMNPV (NPV). (**D**) *M. anisopliae* (LJJ3) versus *S. aureus* (Sa). (**E**) Hierarchical clustering heatmap of all identified DEGs across all infection groups and replicates. In the volcano plots (**B**–**D**), the dashed vertical lines indicate the fold-change thresholds (log2(fold change) = ±1), and the dashed horizontal line indicates the significance threshold (FDR = 0.05; corresponding to −log10(FDR) = 1.30).

Pairwise comparisons between treatment groups identified sets of differentially expressed genes (DEGs). When comparing the *S. aureus* infection to the LdMNPV infection, 308 genes were up-regulated and 263 were down-regulated. The *M. anisopliae* infection showed 623 up-regulated and 413 down-regulated genes relative to the viral infection. Comparing the *M. anisopliae* and *S. aureus* infections revealed 417 up-regulated and 177 down-regulated genes. The overall distribution of these DEGs in each comparison was visualized in volcano plots, while a hierarchical clustering heatmap further illustrated the distinct expression patterns across different samples (Figure 4).

### 3.4. GO Enrichment Analysis Reveals Pathogen-Specific and Common Functional Categories

**Figure 5:**
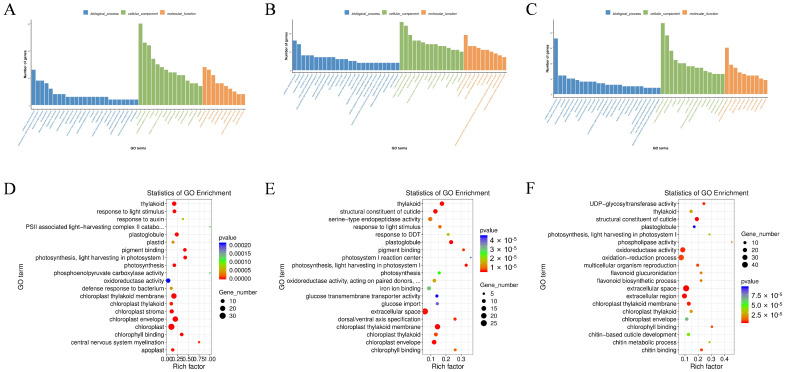
Functional annotation and GO enrichment analysis of differentially expressed genes (DEGs) in response to pathogen infection. (**A**–**C**) GO classification bar charts showing the number of genes associated with biological processes, cellular components, and molecular functions for each comparison: (**A**) *S. aureus* (Sa) vs. CK; (**B**) *M. anisopliae* (LJJ3) vs. CK; (**C**) LdMNPV (NPV) vs. CK. (**D**–**F**) Bubble plots illustrating the top enriched GO terms and their statistical significance: (**D**) *S. aureus* (Sa) vs. CK; (**E**) *M. anisopliae* (LJJ3) vs. CK; (**F**) LdMNPV (NPV) vs. CK. The rich factor indicates the degree of enrichment, while dot size and color represent the number of genes and *p*-value, respectively.

GO enrichment analyses of DEGs in each infection group compared with CK revealed significant functional categorization across the three major ontologies: biological process, cellular component, and molecular function. In the *S. aureus* comparison, terms related to “thylakoid” and “photosynthesis” were notably enriched. For the *M. anisopliae* infection, enrichment was observed in “structural constituent of cuticle” and “serine-type endopeptidase activity”. The LdMNPV infection showed strong enrichment in “chitin metabolic process” and “UDP-glycosyltransferase activity”. The distribution and significance of these enriched terms were further detailed in bubble plots (Figure 5).

**Figure 6:**
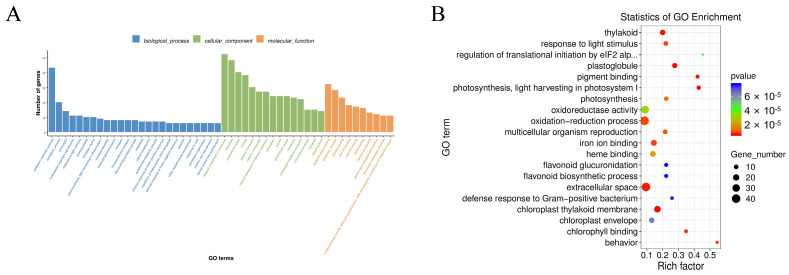
GO functional enrichment analysis of differentially expressed genes (DEGs) between pairwise infection groups. (**A**) GO classification bar chart displaying the number of genes associated with biological processes, cellular components, and molecular functions across all pairwise comparisons. (**B**) Bubble plot illustrating the top enriched GO terms, where the x-axis represents the rich factor, dot size indicates the number of genes, and color gradient represents the *p*-value significance.

GO enrichment analysis of the DEGs identified between infection groups revealed significant enrichment across the three major categories: biological process, cellular component, and molecular function. Within biological processes, “oxidation-reduction process” was the most significantly enriched pathway. For cellular components, “integral component of membrane” and “cytoplasm” showed the greatest enrichment. Among molecular functions, “oxidoreductase activity” was predominant (Figure 6).

### 3.5. LdMNPV Infection Specifically Activates Host Energy Metabolism and Neural Signaling Pathways

**Figure 7:**
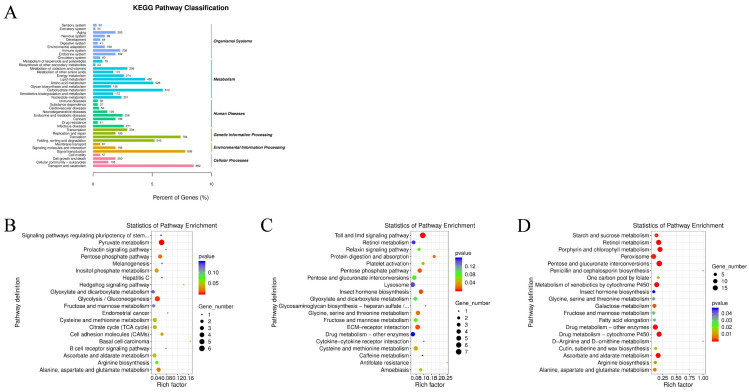
KEGG pathway classification and enrichment analysis of differentially expressed genes (DEGs) in response to pathogen infection. (**A**) KEGG pathway classification bar chart showing the distribution of annotated genes across major functional categories, including Organismal Systems, Metabolism, Human Diseases, Genetic Information Processing, Environmental Information Processing, and Cellular Processes. (**B**–**D**) Bubble plots illustrating the top enriched KEGG pathways for each infection versus control comparison: (**B**) *S. aureus* (Sa) vs. CK; (**C**) *M. anisopliae* (LJJ3) vs. CK; (**D**) LdMNPV (NPV) vs. CK. The rich factor indicates the degree of pathway enrichment, while dot size and color represent the number of genes and *p*-value significance, respectively.

A total of 9247 genes were annotated to 40 KEGG pathways, with the “Transport and catabolism” pathway containing the largest number of genes (882). Pathway enrichment analysis highlighted distinct response profiles depending on the pathogen type. In the *S. aureus* infection group, DEGs were significantly enriched in energy metabolism-related pathways, including “Pyruvate metabolism,” “Pentose phosphate pathway,” and “Glycolysis / Gluconeogenesis”. In contrast, the *M. anisopliae* infection group showed significant enrichment in the “Toll and Imd signaling pathway”, suggesting a robust activation of the innate immune response against fungal infection. The LdMNPV infection group exhibited enrichment primarily in substance metabolism pathways, such as “Starch and sucrose metabolism” and “Porphyrin and chlorophyll metabolism”. These results suggest that while metabolic adjustments are common across all infections, the host mounts a specific immune signaling response primarily against fungal challenges, whereas bacterial and viral infections induce more pronounced metabolic reprogramming (Figure 7).

**Figure 8:**
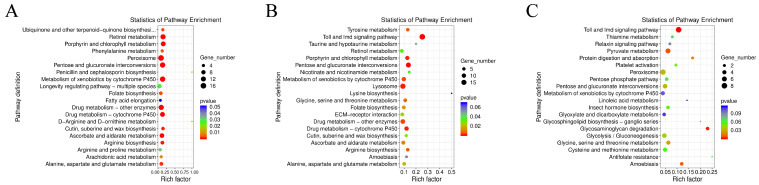
KEGG pathway enrichment analysis of differentially expressed genes (DEGs) between pairwise infection groups. Bubble plots illustrate the top enriched KEGG pathways for each comparison: (**A**) *S. aureus* (Sa) versus LdMNPV (NPV). (**B**) *M. anisopliae* (LJJ3) versus LdMNPV (NPV). (**C**) *M. anisopliae* (LJJ3) versus *S. aureus* (Sa). The x-axis represents the rich factor, dot size indicates the number of genes, and color gradient represents the *p*-value significance.

KEGG pathway enrichment analysis of the DEGs between pairwise infection groups revealed distinct metabolic and immune response profiles depending on the pathogen type. Pairwise comparisons revealed that the “Toll and IMD signaling pathway” was significantly enriched during pathogen infection across all groups. Notably, compared to the other two groups, the virus-infected group showed up-regulation of multiple genes in several energy metabolism-related pathways, including “Nicotinate and nicotinamide metabolism,” “Pyruvate metabolism,” the “Citrate cycle (TCA cycle),” and “Oxidative phosphorylation.” Of particular interest was the significant up-regulation of *hpd* following viral infection, suggesting that the virus may promote energy supply by influencing tyrosine metabolism. Furthermore, the up-regulation of multiple genes within the “Neuroactive ligand-receptor interaction” pathway—such as those encoding trypsin-like proteins and piggyBac transposable element-derived proteins—provides supporting evidence for investigating virus-induced climbing behavior from a neural regulation perspective (Figure 8).

### 3.6. Selected Differentially Expressed Genes Are Validated by qPCR

The expression trends of a subset of seven DEGs, selected for qPCR validation, correlated well with the transcriptomic data, confirming the reliability of the sequencing results (Table 3). Specifically, in the Sa vs. NPV comparison, all seven selected DEGs exhibited consistent down-regulation in both transcriptomic and qPCR analyses, confirming the sequencing trends. While the magnitude of fold change varied—for instance, the log_2_FC for *heparan sulfate 2-O-sulfotransferase pipe* (TRINITY_DN23053_c0_g7) was –2.11 in the transcriptome but –7.01 in qPCR—the direction of change was uniformly negative. Similarly, in the LJJ3 vs. NPV comparison, every gene maintained a down-regulated trend, with qPCR values closely mirroring the RNA-seq patterns, although some quantitative differences were observed (e.g., *alanine aminotransferase 1-like* showed –4.00 vs. –7.42). The small standard deviations (SD) of the qPCR measurements further support the reliability of the validation. These results strongly corroborate the transcriptome data and provide a solid foundation for subsequent analyses.

## 4. Discussion

Through parallel comparison of virus, bacterium, and fungus infections in *L. dispar* larvae, this study conclusively demonstrates within a single host system that the pronounced climbing behavior induced by LdMNPV is a virus-specific behavioral manipulation phenotype, rather than a generalized stress response to severe infection. Behavioral data show that only the virus-infected group exhibited directed, sustained, and significantly elevated upward movement. This finding validates the adaptive significance hypothesis of baculovirus-induced “tree-top disease” [15,17] and establishes a solid phenotypic foundation for subsequent mechanistic investigations.

Transcriptomic analysis revealed that LdMNPV, *S*. *aureus*, and *M. anisopliae* infections all significantly enriched the Toll and IMD signaling pathways, confirming the conserved function of these core immune modules in defending against diverse microbial invaders in *L. dispar*. This result aligns with studies in *Drosophila*, where the Toll pathway primarily responds to fungi and Gram-positive bacteria, the IMD pathway to Gram-negative bacteria [42,43]; both pathways are also engaged in antiviral immunity. However, against this common immune background, LdMNPV infection exhibited far greater transcriptional breadth and specificity than *S. aureus* or *M. anisopliae*. Pairwise comparisons identified 571 and 1036 differentially expressed genes (DEGs) in virus-versus-bacterium and virus-versus-fungus comparisons, respectively, with up-regulated genes substantially outnumbering down-regulated genes. This suggests that viral infection does not simply suppress host functions but actively and selectively remodels the host gene expression network—a pattern consistent with baculovirus hijacking of host transcriptional machinery to facilitate its own replication [44]. This interpretation is also consistent with previous species-specific studies in *L. dispar*, in which antiviral resistance to LdMNPV, fungal infection by Beauveria bassiana, and shifts in symbiotic bacterial composition were all associated with dynamic regulation of innate immune effectors and immune signaling pathways [36,37,38].

KEGG enrichment analysis revealed that, compared with *S. aureus* and *M. anisopliae*, LdMNPV infection significantly enriched multiple energy-metabolism-related pathways, including nicotinate and nicotinamide metabolism, pyruvate metabolism, the citrate cycle (TCA cycle), and oxidative phosphorylation. Several genes encoding key metabolic enzymes within these pathways—such as lactate dehydrogenase, citrate synthase, and ATP synthase subunits—were up-regulated exclusively in the virus-infected group. This pattern was absent in both bacterial and fungal infection groups. Sustained climbing is a characteristically energy-intensive behavior. The observed up-regulation of TCA cycle and oxidative phosphorylation pathways may reflect a state of heightened metabolic activity induced by viral infection to meet the energetic demands of climbing behavior. Recent studies suggest that LdST59 is involved in the redistribution of glucose between different larval tissues after viral infection, and that this shift in energy supply pattern plays an important role in the lethality of LdMNPV to larvae [27]. Similar phenomena have been reported in *Helicoverpa armigera* larvae infected with HearNPV, where the virus alters host lipid metabolism, and these metabolic changes influence climbing behavior [22]. In *Bombyx mori* studies, BmNPV-susceptible strains exhibited significantly increased flux through the phenylalanine–tyrosine metabolic pathway into the TCA cycle following infection, whereas resistant strains did not, suggesting that metabolic reprogramming may be linked to viral proliferation and pathogenicity [24]. Notably, research on behavioral manipulation by entomopathogenic fungi also points to energy metabolism regulation: *Cordyceps militaris* secretes a host-mimetic trehalase, CmTreH1, which degrades trehalose in silkworm hemolymph, inducing a “false hunger signal” and promoting hyperphagia [31]. In contrast to viruses, transcriptomic analyses of fungal-infected *L. dispar* larvae predominantly enrich immune, melanization, and xenobiotic metabolism pathways, with no reported significant enrichment of TCA cycle or oxidative phosphorylation-related genes [38]. Systemic bacterial infections, conversely, are often accompanied by host metabolic suppression and energy depletion, forming a sharp contrast with the metabolic activation characteristic of viral infection [45,46].

Beyond energy metabolism pathways, the “neuroactive ligand-receptor interaction” pathway was the only neural-related pathway significantly enriched exclusively in LdMNPV-infected larvae compared to those infected with *S. aureus* or *M. anisopliae*. This pathway includes genes encoding various neurotransmitters, neuropeptides, and their receptors; altered expression of these genes may affect neural signal transduction efficiency and behavioral output. Histological evidence of baculovirus infection in the insect nervous system exists: BmNPV begins to invade the silkworm brain and ganglia at 48 h post-infection, penetrates internally by 72 h, and infects the entire brain by 120 h, concomitant with morphological loosening of the central complex; RNAi-mediated knockdown of the serotonin receptor Bm5-HT4R enhances behavioral activity in silkworms [47]. Furthermore, HearNPV triggers calcium responses in enteroendocrine cells of *H. armigera*, leading to the release of midgut-derived tachykinin (TK). Released TK activates its receptor (TKR) in the brain, thereby promoting phototaxis and climbing behavior [30]. It is plausible that LdMNPV may similarly infect the nervous system of *L. dispar*, modulate neurotransmitter-related gene expression, and manipulate host behavior. Because the present transcriptomic analysis used whole-body larval homogenates, neural tissue accounted for a relatively small proportion of total biomass; therefore, the enrichment level of neural-related pathways may be underestimated. Our results extend earlier *L. dispar* studies showing dynamic transcriptomic remodeling during the hyperactive stage, the involvement of phototransduction/circadian signaling, and the functional importance of PI3K/AKT–PTEN signaling in terminal climbing behavior [20,21,25].

From an ecological and evolutionary perspective, baculovirus transmission relies on host death and cadaver liquefaction; viral occlusion bodies are then dispersed by wind or rain from elevated positions, making host positioning at plant tops highly advantageous for transmission efficiency [15,17]. Entomopathogenic fungi also depend on airborne conidial dispersal, and certain species have evolved the ability to induce host summit-seeking or phototactic behavior [19,21]. In contrast, most entomopathogenic bacteria—including *S. aureus* used in this study and common species such as *Bacillus thuringiensis*—transmit primarily via peroral ingestion or contact infection [48,49]. The location of host death has a limited impact on the dispersal efficiency of their progeny [50]; consequently, they may lack the evolutionary selective pressure to invest in complex behavioral manipulation mechanisms [51]. In this study, although *S. aureus* infection effectively activated host immune responses, it neither induced climbing behavior nor triggered any virus-like enrichment of energy metabolism or neural pathways. Nevertheless, it must be emphasized that bacteria are not entirely devoid of the ability to influence host behavior. Studies in *Drosophila* have shown that gut microbial metabolites can cross the blood–brain barrier to regulate neuronal activity and locomotor performance [52], and bacterial quorum-sensing signal molecules can crosstalk with the host neuroendocrine system [35]. Specific genetic variants of *Escherichia coli* can enhance aggregation behavior in *C. elegans* [34], and gut microbial metabolites can influence mate preference and feeding decisions in *Drosophila* [53]. However, these effects typically manifest as “byproducts” of commensal–host co-metabolism rather than adaptive strategies specifically evolved by bacteria to promote their own transmission.

## 5. Conclusions

This study demonstrates that the “tree-top disease” phenotype induced by LdMNPV infection is a virus-specific behavioral manipulation. Comparative transcriptomic analysis revealed that LdMNPV infection was associated with extensive reprogramming of the host gene expression network. Notably, in contrast to bacterial and fungal infections, LdMNPV infection specifically correlated with the upregulation of multiple energy metabolism pathways and the neuroactive ligand–receptor interaction pathway. These transcriptional changes may contribute to the enhanced climbing behavior observed in virus-infected larvae. However, we acknowledge that transcriptomic profiling at a single time point (72 h post-infection) limits causal inferences, and future studies with multiple time points will be needed to establish temporal dynamics. Additionally, we acknowledge that poly(A)+ enrichment, while optimal for host transcriptome profiling, may underrepresent bacterial transcripts. Future cross-kingdom comparisons would benefit from total RNA-seq approaches to capture pathogen gene expression dynamics simultaneously. These findings provide candidate targets for subsequent functional validation studies and establish a comparative research foundation for deeper understanding of the molecular mechanisms of pathogen behavioral manipulation.

## Figures and Tables

**Table 1:** Summary of RNA-seq metrics from *L. dispar* transcriptomes.

Sample	CK_1	CK_2	CK_3	Sa_1	Sa_2	Sa_3
Raw_Reads	43,602,676	53,749,502	56,681,550	50,079,460	52,131,104	54,271,364
Raw_Bases	6.54 G	8.06 G	8.50 G	7.51 G	7.82 G	8.14 G
Valid_Reads	42,765,226	52,261,746	55,581,460	49,184,764	51,033,872	53,031,134
Valid_Bases	5.99 G	7.32 G	7.78 G	6.88 G	7.15 G	7.43 G
Valid%	98.08	97.23	98.06	98.21	97.90	97.71
Q20%	98.26	98.35	98.29	98.29	98.27	98.35
Q30%	94.37	94.63	94.50	94.59	94.38	94.59
GC%	47.16	49.25	48.92	52.41	47.74	48.12
*S. aureus*_Reads	5593	6830	7222	1,498,354	1,835,789	1,789,542
*S. aureus* %	0.0131	0.0131	0.0130	3.0464	3.5972	3.3745
*M. anisopliae*_Reads	68,487	79,538	69,259	40,724	71,375	94,653
*M. anisopliae* %	0.1601	0.1522	0.1246	0.0828	0.1399	0.1785
LdMNPV_Reads	17,114	21,753	18,982	30,736	35,448	23,647
LdMNPV %	0.0400	0.0416	0.0342	0.0625	0.0695	0.0446
**Sample**	**LJJ3_1**	**LJJ3_2**	**LJJ3_3**	**NPV_1**	**NPV_2**	**NPV_3**
Raw_Reads	56,183,830	54,906,530	47,558,558	55,877,732	57,088,396	51,368,578
Raw_Bases	8.43 G	8.24 G	7.13 G	8.38 G	8.56 G	7.71 G
Valid_Reads	54,913,522	53,907,414	46,702,590	54,827,504	55,932,562	49,965,302
Valid_Bases	7.69 G	7.54 G	6.54 G	7.68 G	7.83 G	7.00 G
Valid%	97.74	98.18	98.20	98.12	97.98	97.27
Q20%	98.39	98.35	98.40	98.40	98.36	98.43
Q30%	94.82	94.73	94.76	94.73	94.72	94.77
GC%	52.39	53.08	49.30	48.31	51.56	46.46
*S. aureus*_Reads	568	822	862	788	2191	1430
*S. aureus* %	0.0010	0.0015	0.0018	0.0014	0.0039	0.0029
*M. anisopliae*_Reads	1,206,368	952,454	796,372	59,555	45,878	57,790
*M. anisopliae* %	2.1969	1.7668	1.7052	0.1086	0.0820	0.1157
LdMNPV_Reads	26,894	21,653	38,354	14,093,217	12,000,722	11,228,778
LdMNPV %	0.0490	0.0402	0.0821	25.7046	21.4557	22.4732

**Table 2:** Primer sequences for qPCR.

Gene_ID	Forward Primer	Reverse Primer
TRINITY_DN23053_c0_g7	ACAGTATGCCGTCGTAGGAGTC	CTGCTTCACTGCTTCGGATACC
TRINITY_DN27995_c0_g1	TTCGTCGCCGTTCTTGCCTTG	GCCAGCAGCGTAGTCCACAGTA
TRINITY_DN22402_c0_g4	TGGTCAGGTGTTGAGCCGTAGA	CGCCGACGCTATTCTGTTGGT
TRINITY_DN23039_c0_g2	TGGTTGGAGTTGATGCCGTTGG	AGCTCGTCACGCACTCATTCG
TRINITY_DN26771_c2_g2	TGCTGGCAAACGAAAGAGCCA	TCTATGCTCTCTGCGACCCTCA
TRINITY_DN22568_c2_g2	TGGGTGAGGGATGTCGTCTTCT	TGCGTTGCTCTGTGCATACTGT
TRINITY_DN25421_c0_g3	GGAGAAGGTTGGTGGCGTGAAG	ACAGCAATCGCAGTAGCAGGTG

**Table 3:** Verification of differentially expressed genes in transcriptome sequencing by qPCR. (**a**) Sa vs. NPV. (**b**) LJJ3 vs. NPV.

Gene_ID	Annotation	Log_2_FC		SD
		Transcriptome	qPCR	
(**a**)				
TRINITY_DN23053_c0_g7	heparan sulfate 2-O-sulfotransferase pipe [*Spodoptera litura*]	−2.11	−7.01	0.13
TRINITY_DN27995_c0_g1	larval/pupal rigid cuticle protein 66-like [*Trichoplusia ni*]	−3.64	−1.54	0.32
TRINITY_DN22402_c0_g4	alanine aminotransferase 1-like [*Spodoptera litura*]	−4.06	−10.19	2.29
TRINITY_DN23039_c0_g2	purine nucleoside phosphorylase-like isoform X1 [*Spodoptera litura*]	−2.34	−4.09	0.80
TRINITY_DN26771_c2_g2	4-hydroxyphenylpyruvate dioxygenase-like [*Helicoverpa armigera*]	−2.68	−7.3	2.26
TRINITY_DN22568_c2_g2	glutathione S-transferase 2-like [*Bombyx mandarina*]	−2.60	−1.02	0.55
TRINITY_DN25421_c0_g3	low density lipoprotein receptor adapter protein 1-B-like [*Spodoptera litura*]	−2.11	−4.01	0.87
(**b**)				
TRINITY_DN23053_c0_g7	heparan sulfate 2-O-sulfotransferase pipe [*Spodoptera litura*]	−2.68	−8.10	0.47
TRINITY_DN27995_c0_g1	larval/pupal rigid cuticle protein 66-like [*Trichoplusia ni*]	−4.56	−6.14	1.06
TRINITY_DN22402_c0_g4	alanine aminotransferase 1-like [*Spodoptera litura*]	−4.00	−7.42	1.18
TRINITY_DN23039_c0_g2	purine nucleoside phosphorylase-like isoform X1 [*Spodoptera litura*]	−2.27	−3.84	1.50
TRINITY_DN26771_c2_g2	4-hydroxyphenylpyruvate dioxygenase-like [*Helicoverpa armigera*]	−2.76	−3.71	1.58
TRINITY_DN22568_c2_g2	glutathione S-transferase 2-like [*Bombyx mandarina*]	−2.42	−1.83	0.13
TRINITY_DN25421_c0_g3	low density lipoprotein receptor adapter protein 1-B-like [*Spodoptera litura*]	−2.81	−3.08	0.27

Note: FC is fold change, log_2_FC is the log value of FC with 2 as the base. Sa (*S. aureus*) is Bacteria. NPV (LdMNPV) is virus. LJJ3 (*M. anisopliae*) is fungus.

## Data Availability

The raw RNA-seq data have been deposited in the NCBI Sequence Read Archive (SRA) under BioProject accession number PRJNA1402041.
